# Supplementary material for: Genetic diversity of local and introduced cassava germplasm in Burundi using DArTseq molecular analyses
Source: PLoS One. 2022 Jan 24;17(1):e0256002. doi: 10.1371/journal.pone.0256002 (PMC8786168; doi:10.1371/journal.pone.0256002)
Supplement: Attachment — Submitted filename: Response to Reviewers.rtf [file pone.0256002.s009.rtf]

Responses to the Reviewers:  Genetic diversity of local and introduced cassava germplasm in Burundi using DArTseq molecular analyses

Reviewer #1: 

1.	In the abstract the authors mention that the study is aimed at developing a core set of cassava germplasm from the local varieties (Line 30), however no mention of this objective is made in the manuscript text, nor has any analysis been done to this end. The manuscript deals with the genetic diversity present in the landraces and elite lines. Appropriate corrections may be incorporated in the abstract. 

Response: We agree with the reviewer and accordingly any mention of 'core' has been removed from the abstract. 

2.	Discussion on molecular characterization and duplicate identification may elaborated. 

Response: In response to this, results of the discriminant analysis of principal components and principal coordinate analysis have been included. 

3.	Overall, the discussion section may be improved. 

Response: A paragraph on the assessment of population statistics has been included.

4.	Cassava plants were planted in two locations. Did the authors observe any morphological variation among the same plants at the two locations? If yes, authors could indicate the effect of the environment/ soil etc. on the morphological characteristics. 

Response:  No morphological variation among the same genotypes at the two locations has been observed.

5.	Authors have studied cassava landraces from four different sites along with elite introduced germplasm. Can they carry out a population structure analysis for the four different populations and present it graphically? The analysis may bring out important information on the extent of mixing that may have happened in these genotypes over the course of time.

Response: A discriminant analysis of principal components has been included and discussed. 

6.	Authors may also support/depict the genetic diversity and genetic classifications they have observed using Principal Component Analysis (PCA).

Response: This has been included.

7.	Sentence framing and language may be checked in some parts of the introduction and discussion.

Response: This has been addressed


Reviewer #2: 
Diversity Arrays Technology is a high-throughput genotyping method for preferential sampling of gene-rich regions that is achieved through the use of methylation sensitive restriction enzymes.
The authors must use the DArT markers to perform redundancy analysis and assemble the non-redundant sequences for homology. This will provide DArT sequences incorporation into publicly genomics resources. Attribution of putative gene functions can also be done for the non-redundant DArT marker sequences that may aid in identifying markers with putative disease resistance-related functions esp. Cassava Brown Steak Disease (CBSD) and Cassava Mosaic Disease (CMD). 
This will assist in employing the available data in an enhanced way.

Response: Thank you for the comment. All analysis of sequence data, alignments, and SNP calling was carried out by DArT. They performed redundancy analysis and assembled the non-redundant sequences for homology. There were 18,000 SNP markers, making it beyond the scope of this manuscript to assign gene functions. Your comment is appreciated.
